# Complete Genome Sequence of *Agrococcus* sp. Strain SGAir0287, Isolated from Tropical Air Collected in Singapore

**DOI:** 10.1128/MRA.00616-19

**Published:** 2019-08-08

**Authors:** Kenny J. X. Lau, Ana Carolina M. Junqueira, Akira Uchida, Rikky W. Purbojati, James N. I. Houghton, Caroline Chénard, Anthony Wong, Sandra Kolundžija, Megan E. Clare, Kavita K. Kushwaha, Alexander Putra, Nicolas E. Gaultier, Cassie E. Heinle, Balakrishnan N. V. Premkrishnan, Vineeth Kodengil Vettah, Daniela I. Drautz-Moses, Stephan C. Schuster

**Affiliations:** a Singapore Centre for Environmental Life Sciences Engineering, Nanyang Technological University, Singapore; b Departamento de Genética, Instituto de Biologia, Universidade Federal do Rio de Janeiro, Rio de Janeiro, Brazil; Portland State University

## Abstract

Agrococcus sp. strain SGAir0287 was isolated from tropical air samples collected in Singapore. Assembled using single-molecule real-time (SMRT) sequencing and MiSeq reads, the genome consists of one circular chromosome of 3,084,767 bp. The entire genome has 2,870 protein-coding genes, 45 tRNAs, and 3 rRNAs.

## ANNOUNCEMENT

*Agrococcus* species are Gram-positive, nonmotile bacteria classified in the *Actinobacteria* phylum (1). Agrococcus jenensis and its strains 2002-39/1^T^ and ST54 were initially identified as Agromyces spp. but were later assigned to the new genus *Agrococcus* based on their 16S rRNA gene sequence and lack of a mycelial phase (2). Strain 2002-39/1^T^ was isolated from a frozen compost soil, and strain ST54 was isolated from a sandstone building surface. Several new species of *Agrococcus* have been reported since and were found in various environments such as medieval paintings (3), air (4), cheese (5), seaweed (6), soil from cold deserts (7), forests (8), and coal mines (9). Agrococci have an irregular coccoid morphology with characteristic menaquinones and 2,4-diaminobutyric acid cell walls (1). They thrive on tryptic soy agar (TSA; Sigma-Aldrich, USA) at 30°C, with colonies appearing yellow when cultured in light and white when cultured in darkness (1).

*Agrococcus* sp. strain SGAir0287 was isolated from air collected indoors in Singapore (global position system coordinates, 130.048N, 103.791E) using the BioStage single-stage impactor with a QuickTake 30 pump at 28.3 liters/min for 3 min (SKC, Inc., USA). The air was impacted onto Reasoner’s 2A agar (Becton Dickinson, USA), and colonies were further isolated onto TSA plates at 30°C. Genomic DNA was purified using the Wizard genomic DNA purification kit (Promega, USA) according to the manufacturer’s instructions. Library preparation was performed with the SMRTbell template prep kit 1.0 (Pacific Biosciences, USA), followed by single-molecule real-time (SMRT) sequencing on the PacBio RS II platform. Short reads were generated on a MiSeq platform (Illumina, USA) with a 300-bp paired-end run, using whole-genome shotgun libraries constructed with the TruSeq Nano DNA library preparation kit.

A total of 122,093 long reads were used for *de novo* assembly with the Hierarchical Genome Assembly Process version 3 (10). It was then polished in Quiver (10) and error corrected using Pilon version 1.16 (11), including a total of 814,959 MiSeq paired-end reads. The final genome assembly provided one circular contig with 3,084,767 bp (263.16-fold coverage) with a G+C content of 72.99%.

Taxonomic identification was performed using average nucleotide identity (ANI) and 16S rRNA identification, resulting in assignment to the genus *Agrococcus*. ANI was conducted with the microbial species identifier (MiSI) method (12) and had 80.0% identity to Agrococcus lahaulensis DSM 17612, with an alignment fraction of 17%. The 16S rRNA gene analysis using Barrnap version 0.7 (13) and BLASTn (14) against the SILVA database (15) resulted in a match with 98.2% identity to Agrococcus terreus.

The genome was annotated using the NCBI Prokaryotic Genome Annotation Pipeline version 4.2 (16). A total of 2,963 genes were predicted, as follows: 2,870 protein-coding genes; 3 rRNA gene operons, including 5S, 16S, and 23S rRNA; 45 tRNA genes; 3 noncoding RNA genes; and 42 pseudogenes. Functional classification of genes was performed with Rapid Annotations using Subsystems Technology (RAST) (17–19). The annotation results showed that the genome possesses Gram-positive cell wall components but no motility-related genes. It also has menaquinone biosynthesis genes that are characteristic of *Agrococcus* spp. Interestingly, alkanesulfonate assimilation genes were also predicted in its genome. Alkanesulfonate is commonly used in paints, which may explain the presence of *Agrococcus* spp. in paintings (3) and in air near wall paintings in Virgilkapelle (Vienna, Austria) (4).

### Data availability.

The complete genome sequence of *Agrococcus* sp. strain SGAir0287 has been deposited in DDBJ/EMBL/GenBank under the accession number CP027942, and its corresponding Sequence Read Archive (SRA) numbers are SRR8894903 and SRR8894904.
